# Malignant melanoma located in the ureter and gallbladder: A case report and literature review

**DOI:** 10.1097/MD.0000000000037302

**Published:** 2024-02-23

**Authors:** Yanghuang Zheng, Hongjin Shi, Haifeng Wang, Jiansong Wang, Bing Hai, Jinsong Zhang

**Affiliations:** a Department of Urology, the 2nd Affiliated Hospital of Kunming Medical University, Kunming, Yunnan Province, China; b Department of Respiratory Medicine, the 2nd Affiliated Hospital of Kunming Medical University, Kunming, Yunnan Province, China.

**Keywords:** case report, gallbladder, melanoma, ureter

## Abstract

**Rationale:**

Melanoma is one of a common cutaneous malignancy. Currently, metastatic malignant melanoma is difficult to be diagnosed through imaging examinations. Furthermore, the incidence of metastatic melanoma affecting the gallbladder and ureter is exceptionally rare.

**Patient concerns:**

A 54-year-old female was admitted to the hospital with a half-month history of left lower back pain. Correlative examination revealed an occupying lesion in the mid-left ureter and the neck of the gallbladder.

**Diagnoses:**

The patient was initially diagnosed with gallbladder cancer and left ureteral carcinoma based on imaging. Following 2 operations, immunohistochemical staining confirmed the presence of metastatic melanoma involving both the gallbladder and ureter.

**Intervention:**

After multidisciplinary consultation and obtaining consent from the patient and her family, the patient underwent left radical nephroureterectomy, radical cholecystectomy, laparoscopic partial hepatectomy (Hep IV, Hep V), and lymph node dissection of hepatoduodenal ligament.

**Outcomes:**

One month after treatment, the patient imaging showed no disease progression, and at 6 months of follow-up, the patient was still alive.

**Lessons:**

It is difficult to distinguish metastatic melanoma from carcinoma in situ by imaging. In addition, metastatic malignant melanoma lacks specific clinical manifestations and is prone to misdiagnosis, which emphasizes the highly aggressive nature of malignant melanoma.

## 1. Introduction

Melanoma is one of the aggressive and highly metastatic types of cancer caused by excessive proliferation of melanocytes. Melanocytes are neural crest-derived cells found primarily in the basal epidermis and hair follicles, mucosal surfaces, meninges, and choroidal layer of the eye.^[1]^ Melanoma can often develop lymph node metastases and distant metastases. The most common sites of distant metastasis are soft tissues, lungs, liver, skin, and brain.^[2]^ The occurrence of metastatic melanoma in the gallbladder and ureter is infrequent.^[3,4]^

Computed tomography (CT) and Magnetic resonance imaging (MRI) cannot accurately diagnose metastatic melanoma, which can only be clarified by immunohistochemical staining. Metastatic malignant melanoma is often associated with poor prognosis and a short survival cycle. We report a case of malignant melanoma with gallbladder and ureter metastasis, and summarize similar cases in recent years, providing more reference for clinical diagnosis and treatment.

## 2. Case report

**Figure 1.**
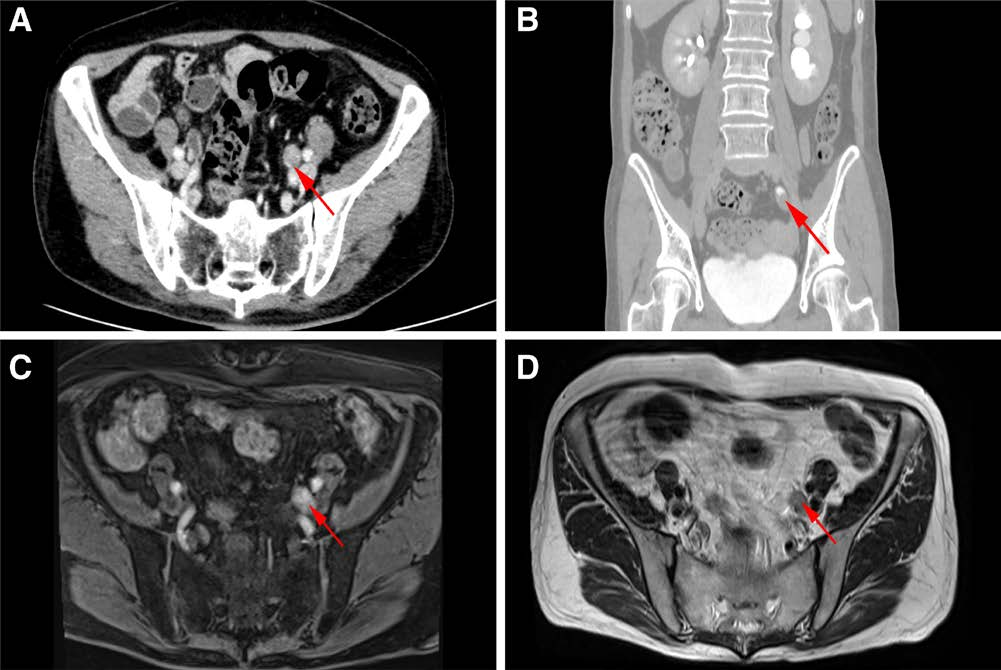
CT urography enhancement phase (A) shows a long-striated enhancing lesion in the left mid-ureter and dilatation of the ureter and renal pelvis above the lesion combined with hydronephrosis; CT urography excretion phase coronal (B). Pelvic MRI: T1WI (C) showed an isotropic T1 markedly intensified signal shadow in the middle part of the left ureter, with a size of about 1.5 × 1.1 × 2.1 cm; T2WI (D) showed a slightly longer T2 signal shadow in the lesion area. The red arrow points to the lesion. CT = computed tomography, MRI = magnetic resonance imaging.

**Figure 2.**
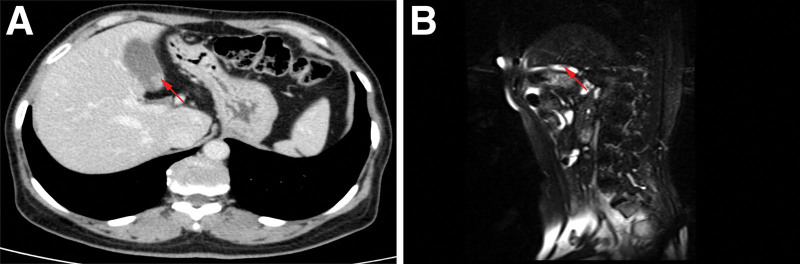
Contrast-enhanced CT (A) of the gallbladder shows irregular nodular enhancement foci in the neck of the gallbladder, measuring approximately 1.2 × 0.9 cm. A long striated lesion is seen in the sagittal position of T2WI of the gallbladder(B). The red arrow points to the lesion. CT = computed tomography.

**Figure 3.**
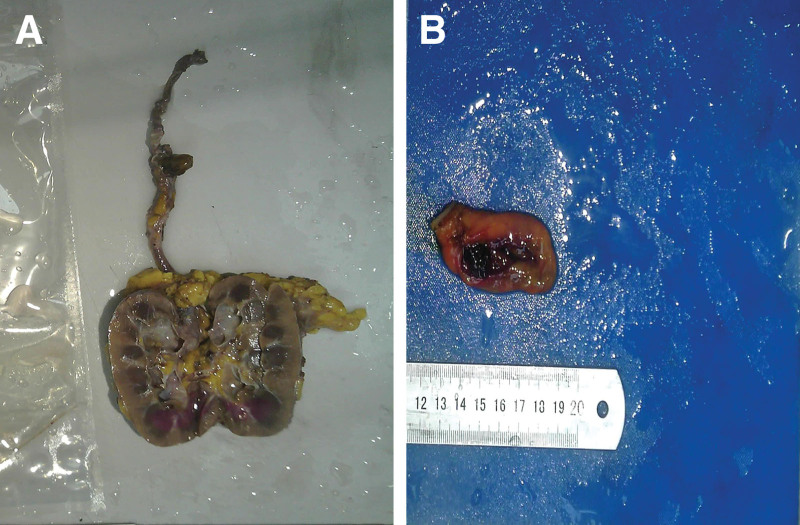
Left renal ureter specimen (A), gallbladder specimen (B).

**Figure 4.**
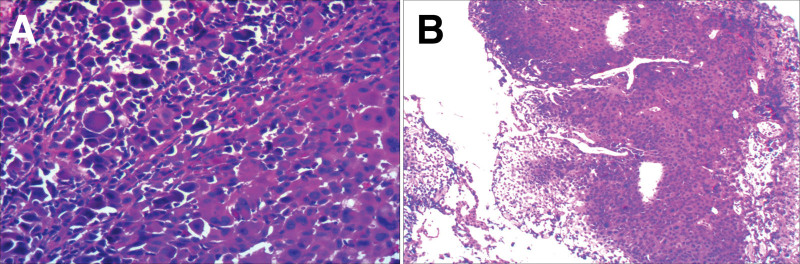
Hematoxylin eosin staining of gallbladder lesion (400×) (A), of ureteral lesion (40×) (B).

**Figure 5.**
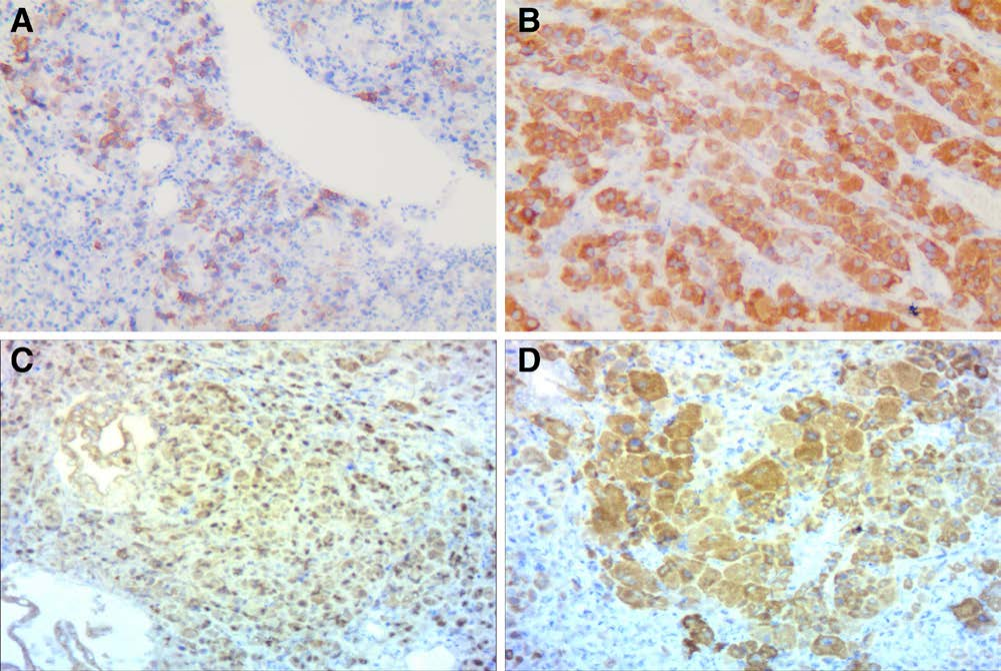
Immunohistochemical staining of ureteral lesions was positive for HBM45 (400×) (A), and Melan-A (400×) (B). Immunohistochemical staining of gallbladder lesions was positive for SOX10 (400×) (C), Melan-A (400×) (D).

A 54-year-old female was admitted to the hospital with a half-month history of left lower back pain. On physical examination, the patient had percussion pain in the left lower back and the rest was unremarkable. She had a previous history of type II diabetes mellitus for 6 years and chronic gastritis for 5 years. Routine blood tests for liver enzymes and creatinine were unremarkable. Tumor markers were unremarkable. Radiologic examination CT urography demonstrated: a strip of intensified shadow in the middle part of the left ureter. Pelvic MRI showed: Slightly long T2 signal shadow was seen in the middle part of the left ureter with obvious enhancement (Fig. 1). Unenhanced and contrast-enhanced CT of the upper abdomen, CT angiography of the portal vein, hepatic artery, and inferior vena cava showed: irregular nodular enhancement foci in the neck of the gallbladder with a size of about 1.2 cm*0.9 cm. MRI of the liver, gallbladder, pancreas, and spleen, and magnetic resonance cholangiopancreatography showed cholecystitis and possible polypoid lesions of the gallbladder (Fig. 2). Three urine cytology tests showed no abnormality. The decision to proceed with left radical nephroureterectomy was made after considering the severity of the left hydronephrosis, reduced glomerular filtration rate in the left kidney, and absence of metabolic function. This decision was reached with the patient and her family consent. The surgical procedure went well, and the subsequent immunohistochemical staining following surgery confirmed the diagnosis of malignant melanoma (Figs. 3A, 4B and 5A and B).

As melanoma is commonly found in the skin, and the patient was found to have malignant melanoma in the ureter, we were highly skeptical that the patient had a preexisting cutaneous melanoma. After detailed questioning and review of the patient condition, it was discovered that the patient had a black nodular lesion on her neck for several decades. We highly suspected that the gallbladder lesion could also be a melanoma. After a multidisciplinary consultation and communication with the patient and the patient family, the decision was made after 1 month postoperatively to management of the gallbladder-occupying lesion. On the day of surgery, laparoscopic cholecystectomy was initially planned; however, a long gray-black polyp was discovered in the neck of the gallbladder intraoperatively (Fig. 3B). During the surgery, the resected gallbladder was placed into an endobag, while ensuring that no bile leakage caused the dissemination of tumor cells in the abdominal cavity. The occupying lesion in the gallbladder was confirmed to be malignant melanoma by rapidly frozen biopsy (Fig. 4A). Following consultation with the patient family members, the surgical approach was modified to include radical cholecystectomy, laparoscopic partial hepatectomy (Hep IV, Hep V), and lymph node dissection of hepatoduodenal ligament. Postoperative immunohistochemical staining reaffirmed the presence of malignant melanoma without evidence of metastasis in resected liver or lymph node tissue. Immunohistochemical staining examination: Melan-A (+); HBM45 (+); SOX10 (+). (Fig. 5C and D). The patient neck lesion was also confirmed as nodular malignant melanoma by dermoscopy and pathology.

One month later, the patient underwent a comprehensive CT scan of the abdomen and chest with contrast enhancement, revealing no evidence of disease progression. We informed patients of the need for genetic testing and emphasized the importance of treating the primary neck lesion and of postoperative adjuvant therapy. However, due to financial constraints, the patient declined our suggestion. As of now, after a 6-month follow-up period, the patient is still alive.

## 3. Discussion and conclusions

Malignant melanoma is a skin tumor with high aggressiveness. Malignant melanoma with metastases to the ureter or gallbladder has been reported in several recent cases (Tables 1 and 2). Samellas et al reported metastatic ureteral melanoma in 1961.^[12]^ The first case of gallbladder malignant melanoma was documented in 1956.^[23]^ Malignant melanoma with concomitant gallbladder and ureteral metastases, however, has not been documented.

**Table 1:** Primary or metastatic malignant melanoma with ureteral metastases.

Authors	Yr	Gender	Age	Primary/Metastatic	2 or more metastases	Clinical approach	Survival
Stoykov et al^[5]^	2022	Male	67	Primary	No	ST, TT	/
March et al^[6]^	2017	Male	51	Metastatic	Yes	ST, IT, TT	/
Macneil et al^[7]^	2016	Male	32	Metastatic	No	ST	>12 M
Khan et al^[8]^	2016	Male	78	Primary	No	ST, TT	Death
Gakis et al^[9]^	2009	Male	46	Metastatic	Yes	ST	/
Senga et al^[10]^	1983	Male	53	Metastatic	No	ST, CHT, IT	>3 M
Judd et al^[11]^	1962	Male	63	Primary	No	ST	>1 M
Samellas et al^[12]^	1961	Male	27	Metastatic	Yes	ST	>2 M

CHT = chemotherapy, IT = immunotherapy, M = Month(s), RT = radiation therapy, ST = surgical treatment, TT = target therapy

**Table 2:** Primary or metastatic malignant melanoma with gallbladder metastases.

Authors	Yr	Gender	Age	Primary/Metastatic	2 or more metastases	Clinical approach	Survival
Bangeas et al^[13]^	2022	Male	68	Metastatic	Yes	ST, RT, TT,	>12M
Jeon et al^[14]^	2021	Male	77	Metastatic	Yes	ST	>52 M
Endo et al^[15]^	2021	Female	78	Uncertain	Yes	ST, IT	>48 M
Hess et al^[16]^	2020	Female	46	Metastatic	Yes	ST, IT	>15 M
D’Urso Vilar et al^[17]^	2020	Female	74	Metastatic	No	ST, IT	>7 M
Saraswat et al^[18]^	2019	Male	81	Metastatic	No	ST, IT	>5 M
Yu et al^[19]^	2018	Male	58	Metastatic	Yes	ST, TT	>26 M
Wang et al^[20]^	2017	Female	63	Primary	Yes	ST, CHT	16 M
Virgilio et al^[21]^	2016	Male	59	Primary	Yes	ST, CHT, IT	>15 M
Giannini et al^[2]^	2016	Male	50	Metastatic	Yes	ST, IT, TT	>6 M
Giannini et al^[2]^	2016	Female	40	Metastatic	No	ST, IT	>6 M
Lo et al^[22]^	2015	Male	59	Primary	No	ST	>8 M

CHT = chemotherapy, IT = immunotherapy, M = Month(s), RT = radiation therapy, ST = surgical treatment, TT = target therapy.

Ultraviolet radiation is the only environmental influence that induces melanoma formation. Ultraviolet radiation causes DNA damage, which stimulates the production of melanocyte-stimulating hormone by keratinocytes in the skin. Melanocyte-stimulating hormone binds to the melanocyte melanocortin receptor-1, which causes the melanocyte to produce and release melanin. B - Raf proto-oncogene (BRAF), neurofibromin-1, and N - RAS mutations are the primary genetic causes of chronically exposed skin-associated melanoma, which is frequently observed on the head, neck, and dorsal region of the upper limbs.^[24,25]^ Long-term patient exposure to ultraviolet radiation may be a contributing factor in the development of melanoma because Yunnan Province in China is situated in a high-altitude environment with strong ultraviolet radiation all year long.

Approximately 25% of melanomas occur in preexisting nevi, and the total number, size, and type of nevi are associated with an increased risk of developing melanoma.^[26]^ The patient had noticed skin lesions on the neck decades ago, which unfortunately were not emphasized. It has been shown that BRAF is mutated in up to 80% of benign nevi, and few benign nevi further develop into melanoma.^[27,28]^ When this usually happens, it is associated with subsequent mutations in key genes, such as telomerase reverse transcriptase or cyclin-dependent kinase inhibitor 2A.

Metastasis of melanoma is often associated with genetic mutations. The RAS/RAF/MEK/ERK signaling cascade, commonly known as the mitogen-activated protein kinase (MAPK) pathway, and the phosphatidylinositol-3-kinase (PI3K)/AKT pathway, are 2 important signaling pathways in melanoma that are frequently abnormally activated as a result of genomic changes.^[29]^ In patients, up to 90% of melanomas caused by chronic ultraviolet radiation exhibit aberrant MAPK pathway activation, with the most common genetic abnormality being a BRAF mutation.^[28,30]^ 80% to 90% of BRAF mutations are V600E (valine to glutamate), while 5% to 12% are V600K (valine to lysine). We hypothesized that the patient might have a BRAF mutation based on the patient regular working environment and the available research. Unfortunately, the patient declined to have genetic testing done.

Metastatic melanoma usually has no specific clinical manifestations. In this case, the patient presented to the hospital with only left lower back pain caused by hydronephrosis. Currently, CT and MRI examinations are still limited in the accurate diagnosis of malignant melanoma, and it is difficult to distinguish the nature of tumors. A definitive diagnosis of melanoma can only be made by immunohistologic staining. Pmel-17/ gp100, MART-1/ Melan A, and tyrosinase derived from melanosomes, S100B derived from cell cytoplasm, MITF and SOX10 derived from nucleus, are common immunohistochemical markers of melanoma. Positive expression of Pmel-17/gp100 and MART-1/Melan A are evidence for melanoma diagnosis.^[31]^

Primary melanoma is mainly treated by surgical resection of involved lesions. Approximately 90% of patients will be cured by surgical treatment, of which 5% may develop metastatic melanoma, which will result in death within 10 years.^[32]^ Such patients with metastatic malignant melanoma had a 4.5 to 8% 10-year survival rate.^[33]^ According to case reports that are currently available, the maximum disease progression-free follow-up for malignant melanoma that invades the ureter is 16 months^[4]^ and patients with metastatic gallbladder melanoma typically live <10 years after diagnosis.^[19]^ Before the era of immunotherapy, surgical treatment was the only treatment that ensured better survival.^[3]^ The development and application of immunotherapeutic drugs have improved the survival cycle of patients with metastatic melanoma compared to the previous period. According to the last case report, surgical treatment of individuals who had isolated melanoma metastases to the gallbladder could extend patients’ lives by 5 years.^[13]^ Besides, Jeon et al reported a case of a melanoma patient with gastric and gallbladder metastases, treated only by radical cholecystectomy and wedge resection of the liver, who survived for more than 52 months without disease progression after surgery.^[14]^ For patients with melanoma who developed ureteral metastases, according to the available literature, the longest survival time for patients treated surgically was more than 12 months without disease progression.^[7]^

The combination of adjuvant therapy and radical surgical removal of the lesion is becoming more popular right now. Interferon, immune checkpoint inhibitors (PD - 1 inhibitors, PD - L1 inhibitors, CTLA - 4 inhibitors), and molecularly targeted medicines (BRAF inhibitors, MEK inhibitors) are often used as adjuvant therapeutic drugs. In 2011, the Food and Drug Administration and the European Medicines Agency approved ipilimumab for the treatment of unresectable metastatic melanoma, and ipilimumab (CTLA - 4 inhibitors) showed a significant over-survival benefit compared to the gp100 vaccine.^[34]^ In 2017, research showed that nivolumab (PD - 1 inhibitors) adjuvant therapy resulted in significantly longer recurrence-free survival (70.5% vs 60.8%) and a lower rate of grade 3 or 4 adverse events in patients with resection of stage IIIB, IIIC, or IV melanoma with a minimum 18-month follow-up compared to Ipilimumab adjuvant therapy.^[35]^ Nivolumab was approved by the Food and Drug Administration as an adjuvant medication for patients with high-risk resected melanoma based on the research early findings. In addition, the combination of ipilimumab and nivolumab for the treatment of advanced melanoma resulted in a 5-year overall survival rate of 52%.^[36]^ BRAF-targeted medications, such as vemurafenib, can be utilized as the first line of treatment for advanced metastatic illness in individuals with demonstrable BRAF mutations.^[37]^ Compared to BRAF-targeted drugs alone, adjuvant therapy using BRAF and MEK inhibitors dramatically improves the long-term prognosis of high-risk melanoma patients with BRAF mutations.^[38]^ Post-surgical combined adjuvant treatment of melanoma patients with ureteral metastases has not been documented with more than 16 months of follow-up.^[4]^ For patients with malignant melanoma involving both gallbladder and ureter, no survival cycle has been reported. Up to now, the patient we reported has survived for 6 months without disease progression.

In conclusion, we reported a case of malignant melanoma with metastasis to the gallbladder and ureter. Imaging examinations for diagnosing metastatic malignant melanoma remain challenging. The prognosis for patients with metastatic melanoma is unfavorable. The highly aggressive nature of melanoma cannot be disregarded. Although surgical treatment combined with adjuvant therapy can offer survival benefits, the financial burden should also be considered.

## Author contributions

**Data curation:** Yanghuang Zheng, Hongjin Shi, Haifeng Wang, Jiansong Wang.

**Writing – original draft:** Yanghuang Zheng.

**Writing – review & editing:** Bing Hai, Jinsong Zhang.

## References

[1] Kanitakis J. Anatomy, histology and immunohistochemistry of normal human skin. Eur J Dermatol. 2002;12:390–9; quiz 400. 12095893

[2] Giannini I Cutrignelli DA Resta L. Metastatic melanoma of the gallbladder: report of two cases and a review of the literature. Clin Exp Med. 2016;16:295–300. 25929736 10.1007/s10238-015-0353-6

[3] Marone U Caraco C Losito S. Laparoscopic cholecystectomy for melanoma metastatic to the gallbladder: is it an adequate surgical procedure? Report of a case and review of the literature. World J Surg Oncol. 2007;5:141. 18072972 10.1186/1477-7819-5-141 PMC2233626

[4] Acikalin A Bagir E Karim S. Primary melanoma of the urinary tract; clinicopathologic and molecular review of a case series. Pathol Res Pract. 2020;216:153095. 32825962 10.1016/j.prp.2020.153095

[5] Stoykov B Velev D Ali A. Adrenal and ureteral metastasis of malignant melanoma: a case report and review of the literature. Urol Case Rep. 2022;45:102286. 36438454 10.1016/j.eucr.2022.102286 PMC9684693

[6] March B Calopedos RJS Latif E. Ureteric obstruction from malignant melanoma in both right double moiety and left single moiety ureters. Urology. 2017;103:e7–8. 28216451 10.1016/j.urology.2017.02.021

[7] Macneil J Hossack T. A case of metastatic melanoma in the ureter. Case Rep Urol. 2016;2016:1853015. 27818830 10.1155/2016/1853015 PMC5080517

[8] Khan M O’Kane D Du Plessis J. Primary malignant melanoma of the urinary bladder and ureter. Can J Urol. 2016;23:8171–5. 26892061

[9] Gakis G Merseburger AS Sotlar K. Metastasis of malignant melanoma in the ureter: possible algorithms for a therapeutic approach. Int J Urol. 2009;16:407–9. 19416402 10.1111/j.1442-2042.2008.02238.x

[10] Senga Y Furuhata A Murakami M. [A case of malignant melanoma of the penis]. Hinyokika Kiyo. 1983;29:1079–83. 6677141

[11] Judd RL. Melanoma of the ureter: a case report. J Urol. 1962;87:805–7. 14452693 10.1016/S0022-5347(17)65051-1

[12] Samellas W Marks AR. Metastatic melanoma of the urinary tract. J Urol. 1961;85:21–3. 13745898 10.1016/S0022-5347(17)65276-5

[13] Bangeas PI Bekiaridou A Tsolakidis A. Role of minimally invasive surgery in the treatment of gallbladder metastatic melanoma. A review of the literature and a case report. Cancer Rep (Hoboken). 2022;5:e1549. 34981676 10.1002/cnr2.1549 PMC9327651

[14] Jeon HJ Kwon HJ Kim SG. Malignant melanoma of the gallbladder: a case report and literature review. Ann Hepatobiliary Pancreat Surg. 2021;25:445–9. 34402451 10.14701/ahbps.2021.25.3.445 PMC8382871

[15] Endo M Yano S Asano H. Long-term survival with a rare advanced primary gastrointestinal malignant melanoma treated with laparoscopic surgery/immune checkpoint inhibitor. Acta Med Okayama. 2021;75:231–8. 33953431 10.18926/AMO/61906

[16] Hess GF Glatz K Rothschild SI. Malignant melanoma metastasis in the gallbladder. A case report of an unusual metastatic site. Int J Surg Case Rep. 2020;75:372–5. 32980711 10.1016/j.ijscr.2020.09.116 PMC7522582

[17] D’Urso Vilar GG Iriarte F Speisky D. Isolated gallbladder metastasis of melanoma: case report. Int J Surg Case Rep. 2020;71:311–4. 32485637 10.1016/j.ijscr.2020.04.086 PMC7264460

[18] Saraswat NB DeVoe WB. Metastatic melanoma of the gallbladder presenting as polyp in acute cholecystitis. J Surg Case Rep. 2019;2019:rjz324. 31844512 10.1093/jscr/rjz324 PMC6905303

[19] Yu Z Quiroz E Shen Y. Pathological complete response induced by neoadjuvant treatment using BRAF and MEK inhibitors in a patient with unresectable BRAF V600E-mutant malignant melanoma of the gallbladder. Onco Targets Ther. 2018;11:8723–8. 30584330 10.2147/OTT.S177111 PMC6287667

[20] Wang JK Su F Ma WJ. Primary malignant melanoma of the gallbladder with multiple metastases: a case report. Medicine (Baltim). 2017;96:e8793. 10.1097/MD.0000000000008793 PMC5704886 29145341

[21] Virgilio E Scorsi A Amodio PM. Primary malignant melanoma of the gallbladder: an outstandingly rare tumor. Clin Exp Med. 2016;16:479–80. 26183612 10.1007/s10238-015-0378-x

[22] Lo AA Peevey J Lo EC. Isolated gallbladder intramucosal metastatic melanoma with features mimicking lymphoepithelial carcinoma. Int J Surg Pathol. 2015;23:409–13. 26041740 10.1177/1066896915588932

[23] Kazmann HA Zukaukas CL. Malignant melanoma of the gallbladder. Am J Surg. 1956;92:469–71. 13354875 10.1016/s0002-9610(56)80125-6

[24] Curtin JA Fridlyand J Kageshita T. Distinct sets of genetic alterations in melanoma. N Engl J Med. 2005;353:2135–47. 16291983 10.1056/NEJMoa050092

[25] Ribas A Puzanov I Dummer R. Pembrolizumab versus investigator-choice chemotherapy for ipilimumab-refractory melanoma (KEYNOTE-002): a randomised, controlled, phase 2 trial. Lancet Oncol. 2015;16:908–18. 26115796 10.1016/S1470-2045(15)00083-2 PMC9004487

[26] Leonardi GC Falzone L Salemi R. Cutaneous melanoma: from pathogenesis to therapy (Review). Int J Oncol. 2018;52:1071–80. 29532857 10.3892/ijo.2018.4287 PMC5843392

[27] Shain AH Bastian BC. From melanocytes to melanomas. Nat Rev Cancer. 2016;16:345–58. 27125352 10.1038/nrc.2016.37

[28] Gray-Schopfer V Wellbrock C Marais R. Melanoma biology and new targeted therapy. Nature. 2007;445:851–7. 17314971 10.1038/nature05661

[29] Shimizu T Tolcher AW Papadopoulos KP. The clinical effect of the dual-targeting strategy involving PI3K/AKT/mTOR and RAS/MEK/ERK pathways in patients with advanced cancer. Clin Cancer Res. 2012;18:2316–25. 22261800 10.1158/1078-0432.CCR-11-2381

[30] Wang YF Jiang CC Kiejda KA. Apoptosis induction in human melanoma cells by inhibition of MEK is caspase-independent and mediated by the Bcl-2 family members PUMA, Bim, and Mcl-1. Clin Cancer Res. 2007;13:4934–42. 17652623 10.1158/1078-0432.CCR-07-0665

[31] Timar J Ladanyi A. Molecular pathology of skin melanoma: epidemiology, differential diagnostics, prognosis and therapy prediction. Int J Mol Sci. 2022;23:5384. 35628196 10.3390/ijms23105384 PMC9140388

[32] Dye DE Medic S Ziman M. Melanoma biomolecules: independently identified but functionally intertwined. Front Oncol. 2013;3:252. 24069584 10.3389/fonc.2013.00252 PMC3781348

[33] Karakousis G Gimotty PA Bartlett EK. Thin melanoma with nodal involvement: analysis of demographic, pathologic, and treatment factors with regard to prognosis. Ann Surg Oncol. 2017;24:952–9. 27807729 10.1245/s10434-016-5646-9 PMC5555768

[34] Hodi FS O’Day SJ McDermott DF. Improved survival with ipilimumab in patients with metastatic melanoma. N Engl J Med. 2010;363:711–23. 20525992 10.1056/NEJMoa1003466 PMC3549297

[35] Weber J Mandala M Del Vecchio M. Adjuvant Nivolumab versus ipilimumab in resected stage III or IV melanoma. N Engl J Med. 2017;377:1824–35. 28891423 10.1056/NEJMoa1709030

[36] Larkin J Chiarion-Sileni V Gonzalez R. Five-year survival with combined nivolumab and ipilimumab in advanced melanoma. N Engl J Med. 2019;381:1535–46. 31562797 10.1056/NEJMoa1910836

[37] Nenclares P Ap Dafydd D Bagwan I. Head and neck mucosal melanoma: the United Kingdom national guidelines. Eur J Cancer. 2020;138:11–8. 32829104 10.1016/j.ejca.2020.07.017

[38] Long GV Hauschild A Santinami M. Adjuvant dabrafenib plus trametinib in stage III BRAF-mutated melanoma. N Engl J Med. 2017;377:1813–23. 28891408 10.1056/NEJMoa1708539

